# Context-specific activation of hippocampus and SN/VTA by reward is related to enhanced long-term memory for embedded objects

**DOI:** 10.1016/j.nlm.2015.11.018

**Published:** 2016-10

**Authors:** Eleanor Loh, Dharshan Kumaran, Raphael Koster, David Berron, Ray Dolan, Emrah Duzel

**Affiliations:** a Wellcome Trust Centre for Neuroimaging, University College London, London WC1N 3BG, United Kingdom; b Institute of Cognitive Neuroscience, University College London, London WC1N 3AR, United Kingdom; c Institute of Cognitive Neurology and Dementia Research, Otto-von-Guericke University, 39120 Magdeburg, Germany; d Berlin School of Mind and Brain, Humboldt University, 10099 Berlin, Germany; e Max Planck UCL Centre for Computational Psychiatry and Ageing Research, University College London, London WC1B 5EH, United Kingdom

**Keywords:** SN/VTA, substantia nigra/ventral tegmental area, DG, dentate gyrus, Hippocampus, Reward, Dopamine, Pattern-separation, Memory, Context

## Abstract

Animal studies indicate that hippocampal representations of environmental context modulate reward-related processing in the substantia nigra and ventral tegmental area (SN/VTA), a major origin of dopamine in the brain. Using functional magnetic resonance imaging (fMRI) in humans, we investigated the neural specificity of context-reward associations under conditions where the presence of perceptually similar neutral contexts imposed high demands on a putative hippocampal function, pattern separation. The design also allowed us to investigate how contextual reward enhances long-term memory for embedded neutral objects. SN/VTA activity underpinned specific context-reward associations in the face of perceptual similarity. A reward-related enhancement of long-term memory was restricted to the condition where the rewarding and the neutral contexts were perceptually similar, and in turn was linked to co-activation of the hippocampus (subfield DG/CA3) and SN/VTA. Thus, an ability of contextual reward to enhance memory for focal objects is closely linked to context-related engagement of hippocampal–SN/VTA circuitry.

## Highlights

• We tested if a rewarding context improved memory for embedded objects.
• Pattern-separation demands associated with context discrimination were manipulated.
• Contextual reward improved object memory in the similar condition alone.
• Improved memory was linked to context-related activation of the DG/CA3 and SN/VTA.
• SN/VTA engagement may determine whether memories are improved by contextual reward.

## 1 Introduction

Learning which contexts are associated with reward value is thought to depend on functional interaction between the hippocampus and the SN/VTA (Lisman et al., 2011, Luo et al., 2011). An outstanding question concerns whether a rewarding context can influence memory for the events embedded within it. Reinforcement learning theory would posit that objects embedded into a context should not acquire any reward-related benefits if the reward is already fully predicted by the context (Kamin, 1969, Rescorla and Wagner, 1972). In contrast, the neurobiology of hippocampal–SN/VTA interactions would theoretically predict such contextual memory benefits. Specifically, contextual activation of the hippocampus can lead to a tonic up-regulation of SN/VTA activity, thereby influencing dopamine release to co-occurring events (Goto & Grace, 2008). Additionally, dopamine release in response to a rewarding context can up-regulate protein-synthesis in hippocampal neurons, thereby affecting plasticity for embedded events that occur in close temporal proximity to dopamine release (so-called synaptic tag-and-capture; see Redondo & Morris, 2011, for review).

Given the importance of the hippocampus in regulating SN/VTA activity (Lisman and Grace, 2005, Luo et al., 2011), it is conceivable that contextual reward effects on memory might be particularly strong when learning and retrieving context-reward associations poses high demands on hippocampal processing. This is likely to be the case when it is necessary to discriminate between perceptually similar contexts (Graham et al., 2006, Kesner, 2007, Lee et al., 2005). Indeed, the formation of distinct memory representations for similar environments depends on the ability of the dentate gyrus (DG) to perform pattern separation on inputs from the entorhinal cortex, resulting in distinct representations that are maintained at the level of the hippocampal subfield CA3 (Bakker et al., 2008, Bonnici et al., 2012, Graham et al., 2006, Kesner, 2007, Kesner, 2013, Marr, 1971, McNaughton and Morris, 1987, Mundy et al., 2013, Neunuebel and Knierim, 2014, O’Keefe and Nadel, 1979). Therefore, intact learning about rewarding contexts that are perceptually similar should depend on neural representations that are supported by the CA3 and SN/VTA. Indeed, a pathway linking CA3 with the SN/VTA has recently been reported (Luo et al., 2011). Rewarding contexts whose discrimination involves this projection from the CA3 to the SN/VTA may thus exert particularly strong drive on the SN/VTA, compared to rewarding contexts that lead to a reward-related SN/VTA response via other neural means.

**Fig. 1:**
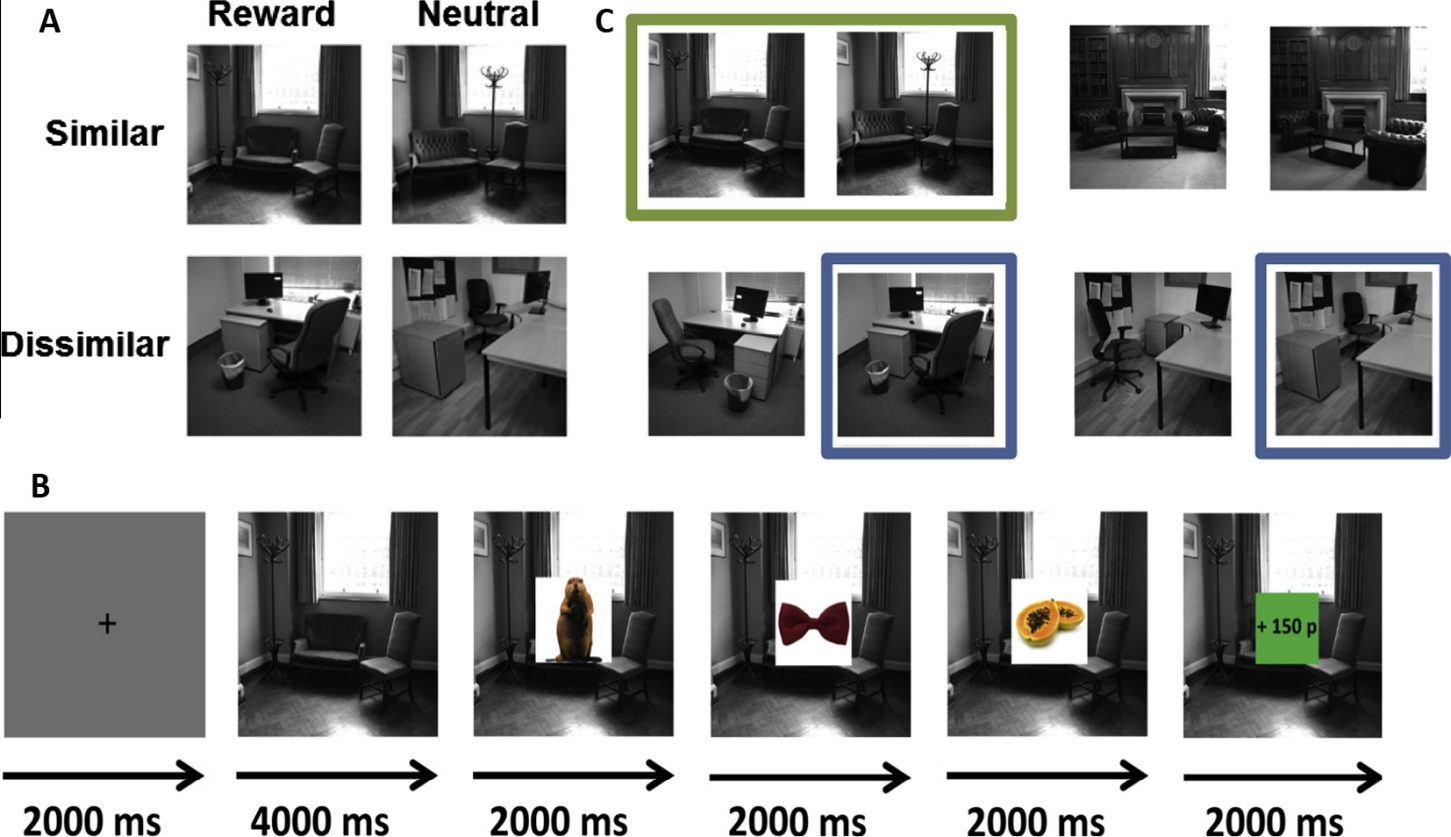
Experimental design (A) Examples of four unique context stimuli seen by a single participant. Context stimuli are divided into a similar pair and a dissimilar pair, where one context picture in each pair is rewarded and the other is neutral (producing four experimental context conditions: similar-reward, similar-neutral, dissimilar-reward, dissimilar-neutral). Because discrimination of the similar context pictures should theoretically place demands on hippocampal pattern separation (see main text for more detail), reward-related responding in the similar condition in particular should rely on pattern separated context representations in the CA3 subfield of the hippocampus. (B) Trial sequence for the encoding-phase, performed in scanner. Reinforcement–neutral objects were presented with reinforcement-predicting context pictures in the background. On each trial, participants made semantic judgements to each object as it came onscreen (indicating if it was a man-made or natural object). The context picture on each trial determined whether there was money available to be won on the trial or not; if money was available, participants would win +50p for each object to which they had made a quick and accurate response. The object stimuli were subject to a surprise memory test after a 5 day delay. (C) In order to control for stimulus-specific effects relating to the context stimuli, the exact four context stimuli seen by each participant was randomly counterbalanced across all participants. Four different similar context stimuli pairs were created by altering the positions of furniture within four different rooms (two offices and two living rooms). For each participant, a similar and dissimilar pair of contexts were assembled by choosing one similar context pair (e.g. two similar living rooms, outlined in green), as well as one picture from each of the two context pairs from the other room category (e.g. two office context pictures, one from each of the office context pairs, outlined in blue).

We hypothesized that the ability to discriminate a rewarding context from a similar but neutral context would invoke co-activation of DG/CA3 and SN/VTA. We set out to determine (i) whether a rewarding context benefitted memory for embedded objects, (ii) the extent to which such a benefit related to a co-activation of DG/CA3 and SN/VTA, and (iii) the extent to which such a benefit was modified by demands on contextual pattern separation. Participants underwent context conditioning for a pair of similar and dissimilar pictures, where one context picture in each pair was associated with reward and the other was associated with a neutral outcome (Fig. 1A). During fMRI scanning, pictures of objects were superimposed on these context pictures (Fig. 1B), and incidental memory for the objects was tested after a five-day delay. We employed high-resolution fMRI alongside specialized spatial normalization protocols, in order to determine if any such mnemonic effects were specifically related to co-engagement of the DG/CA3 and SN/VTA.

## 2 Materials and methods

### 2.1 Participants

Twenty-seven adults participated in the experiment (9 male; age range 19–31 years; mean = 22.85, SD = 3.08 years). Two participants were excluded from both behavioural and MRI analyses on the basis of poor overall memory (*d*′ < 0.3), and one further participant was excluded from MRI analysis on the basis of poor MRI coverage. Overall, 25 subjects were included in behavioural analysis, and 24 subjects were included in the general fMRI analysis. In the brief behavioural analysis of ‘know’ rates only, a further 2 subjects were removed for having negative corrected know rates (indicating more false alarms than hits in ‘know’ memory judgments), in additional to the two participants that had been excluded on the basis of poor overall memory. All participants were healthy, right-handed, and had normal or corrected-to-normal visual acuity. None of the participants reported a history of neurological or psychiatric conditions, or significant medications. All experiments were run with each participant’s written informed consent and according to the local ethics clearance (University College London, London, UK).

### 2.2 Experimental design and task

The task was divided into three stages: a context-conditioning stage, a context-dependent object encoding stage, and a memory test. In the first stage (context conditioning stage: not scanned), participants were trained to associate 4 unique context stimuli (i.e. background pictures depicting an indoor environment; Fig. 1A) with either the presence or absence of monetary reward, by performing a box-probe task in which the background context indicated whether money was available to win on that trial or not. Each participant saw a pair of similar context pictures and a pair of dissimilar ones in the experiment, with one picture in each pair being rewarded and the other not. Participants were instructed that they would see 4 unique context stimuli, grouped into pairs according to the type of room depicted (office or living room), and that one picture in each pair would be rewarded. The exact stimuli used and their assignment to the similarity and valence conditions was counterbalanced across all subjects (see later section on Stimuli for more details). The background context stimulus was shown onscreen for 4000 ms, after which a blue box appeared (with a jittered onset of 1100–1600 ms) briefly in one of the four quadrants of the picture. Participants were instructed to press a button when the blue box appeared, and, if money was available to win on that trial (i.e. as indicated by the background context picture), then they would win +100p if their response was sufficiently quick. The background context stimulus was displayed for the entire length of each trial (between 6000 and 7000 ms), and participants viewed a blank screen with a fixation cross during the inter-trial interval (ITI; 1000 ms). Each context stimulus was presented 30 times, and the context–reinforcement relationships were held constant for each participant throughout the entire experiment. Participants learned through trial-and-error which context stimuli predicted reward and which predicted the absence of reward. This session lasted for roughly 20 min in total, and verbal report following this training confirmed that all participants had learned these associations with full accuracy. Participants were told to respond on every trial regardless of whether they thought money was available to win or not, and response thresholds were set according to each participant’s performance in an earlier box-probe thresholding task (in which they made speeded responses to the appearance of a box probe, without any context stimuli in the background; the mean + 1 SD response time in the thresholding task was used as the response threshold during the context conditioning stage). This stage was performed on a desktop computer just before participants entered the MRI scanner for the second stage of the experiment.

In stage 2 (context-dependent object encoding stage: scanned), participants saw the same 4 unique context stimuli, while making semantic judgments to object stimuli that were superimposed on top (Fig. 1B). On each trial, the context was presented onscreen for 4000 ms, after which three object pictures were presented for 2000 ms each, one after the other, with the context image remaining in the background. Participants made speeded semantic judgments to each object, indicating if they were man-made or natural. As in stage 1, the background context stimuli determined whether monetary reward was available or not. If monetary reward was available on a given trial, participants were able to win money by being quick and accurate in their semantic judgments of each object (+50p per object). The threshold for a quick response was again adjusted for each participant, according to their performance on an earlier thresholding task in which they made quick semantic judgments to practice object pictures without any co-presented background stimuli (mean + 1 SD response time in the thresholding task was again used as the response threshold for this stage). At the end of 50% of all trials, participants were provided with feedback specifying how much money they had won on that trial, and on the other half of trials no feedback was provided (i.e. a question mark was displayed). This standard procedure was adopted to allow us to de-correlate the presentation of reward-predicting context stimuli from the receipt of monetary reward in the fMRI analysis, and participants were told that they would still receive the money won on trials where the feedback was not directly shown. Participants were instructed to perform as well as they could on all trials, regardless of reinforcement. To further encourage them to do so, slow or incorrect responses on neutral trials had a 25% chance of incurring a small loss of −5p. As before, the background context stimuli stayed onscreen in the background throughout the entire trial, and participants viewed a blank screen with a fixation cross during the ITI (2000 ms). Participants saw 288 trial-unique object stimuli (144 man-made, 144 natural) during this stage of the experiment, together with the 4 unique context images (each repeated 24 times), for a total of 96 trials. This session lasted approximately 23 min in total.

Stage 3 of the experiment (object memory test: not scanned) was conducted 5 days later. The five-day delay was based on pilot experiments, which indicated that we were likely to observe ceiling effects when examining memory after a one-day delay. Participants saw 428 objects onscreen (288 of which they had seen before in Stage 2 of the experiment, 140 of which were new), and for each object had to decide whether it was old (if they had seen it before in the experiment) or new. If the object was deemed to be old, participants were then asked if they “Knew” or “Remembered” the object. Following this judgment, participants were asked to indicate whether their memory for the object was “strong” or “weak”. We followed standard procedures in instructing participants about remember and know judgments (Tulving, 1985); specifically, participants were instructed to give a ‘Remember’ response if they could recollect any other details from when they had initially seen the object, and were instructed to respond with ‘Know’ if they could not recollect any other details about the object, and merely had a sense of it being familiar. Detailed instructions regarding this distinction were relayed to participants, along with examples of such memories as one would encounter them in daily life, to ensure that participants understood how they should respond in the task. All memory measures were corrected for false alarm rates, and *d*′ [*Z*(hit rate) − *Z*(false alarm rate)] was used to index recognition memory. Overall, participants were compensated for participation in the experiment at a rate of £6/h for behavioural tasks and £10/h for MRI. Participants also received a proportion of the total amount of money that they had won in the experiment (in stage 1 and 2 of the experiment). On average, subjects won money for an average of 88.22% of the objects for which money was available to win (SD = 9.34%), which came to mean winnings of roughly £22, in addition to the compensation for time.

A 2 × 2 (Similarity × Valence) Factorial design was employed for the Context images, and behavioural measures (response speeds, recognition accuracy) were analyzed with a 2 × 2 (Similarity × Valence) repeated-measures factorial ANOVA.

### 2.3 Stimuli

Context stimuli were specifically created for this experiment, and consisted of grayscale pictures of offices and living rooms with no human beings in them (Fig. 1C). The similar pictures were created by changing the position of furniture within room, without adding or removing any elements in the scene. Dissimilar context stimuli consisted of two pictures from two different rooms (belonging to the same category, e.g. two different offices). Four similar context-picture pairs were created for this experiment (2 living room and 2 office). In order to eliminate stimulus-specific effects relating to the context stimuli, the exact context stimuli used for each participant (and their assignment within the factorial design) was counterbalanced across the entire group, by drawing different similar–dissimilar permutations of the context stimuli from the original four similar context-pairs (Fig. 1C). Each participant saw four unique context stimuli, repeated throughout the experiment. Object stimuli consisted of colour images assembled from the Brady, Konkle, Alvarez, and Oliva (2008) database of object stimuli as well as some additional images from the internet, and were balanced in terms of semantic category (man-made versus natural).

### 2.4 fMRI data acquisition and preprocessing

Data was acquired using a 3T Quattro Siemens scanner (Siemens Healthcare, Erlangen, Germany) operated with a 32-channel head coil. Functional data was acquired using a three-dimensional gradient-echo T2^*^-weighted echo-planar imaging (EPI) sequence (TR = 62.5 ms/slice, 2.5 s/volume, TE = 30 ms, flip angle = 15°), covering a partial volume that included the hippocampus, striatum and midbrain (40 oblique axial slices per volume acquired in ascending order; field of view = 192 mm; slab angled at −45° in the anteroposterior axis; spatial resolution = 2 mm isotropic), using a functional sequence that was optimized for the hippocampus and midbrain (Lutti, Thomas, Hutton, & Weiskopf, 2013; see Supplementary Fig. 2A for mean coverage). Respiration and heart rate were recorded using a breathing belt and pulse oximeter, and used to correct for respiration- and heartbeat-related artefacts (Hutton et al., 2011). Individual field maps were also acquired using the standard manufacturer’s double echo gradient echo field map sequence (TE = 10.0 and 12.46 ms, TR 1020 ms; matrix size, 64 × 64; 64 slices, spatial resolution = 3 × 3 × 3 mm), to allow for distortion correction using the SPM Fieldmap toolbox (Hutton et al., 2002). Multiparameter structural images (including T1-weighted and magnetization-transfer contrasts; spatial resolution = 1.3 mm isotropic) were acquired using established protocols (Weiskopf & Helms, 2008). These high-resolution imaging protocols allow us to localize any observed neural activations to specific hippocampal subfields, though it precludes reliable differentiation of the DG and CA3 regions. As such, the DG and CA3 were treated as a single combined region in our analysis. All data analysis (aside from spatial normalization) was conducted in SPM8 (Wellcome Trust Centre For Neuroimaging, London, UK). Preprocessing included bias correction, realignment, unwarping (using individual fieldmaps), and smoothing with a 4 mm Gaussian kernel. Standard spatial normalization steps were omitted during preprocessing, in lieu of the specialized protocols.

### 2.5 Spatial normalization to allow for hippocampal subfield localization

Spatial normalization was conducted using the programme Advanced Normalization Tools (ANTs; Avants, Tustison, Wu, Cook, & Gee, 2011), which implements SyN (symmetric normalization), a powerful diffeomorphic registration algorithm (Klein et al., 2009) commonly used for hippocampal subfield localization. Using this procedure, a group template brain is first constructed using the structural T1-weighted images of all participants, and transformations mapping between each participants native space and the group template are then calculated, guided by user-specified anatomical landmarks that are marked in the group and individual participant spaces (bilateral landmarks used: anterior-most edge of the hippocampus; posterior hippocampus; superior, inferior, medial and lateral borders of the hippocampus on the first coronal slice where the uncus is clearly visible; superior, inferior and middle borders of the SN/VTA). Spatial normalization was then implemented by using these transformations to bring the first-level statistical maps (first level contrasts; see later section for detail) from each participant into the group template space. Importantly, this procedure allows for inverse mapping of group-level results clusters back into the native space of individual participants, which allows us to verify that group-level hippocampal voxels in DG/CA3 did indeed map onto the DG/CA3 hippocampal subregion in all individual participants’ anatomical scans.

### 2.6 Voxel-based fMRI analysis

A single first-level General Linear Model was employed to examine all neural activations relating to the contexts, objects, and overall memory. The model included four separate regressors corresponding to the background contexts of our 2 × 2 factorial design (i.e. Similar-Rewarded, Similar-Neutral, Dissimilar-Rewarded and Dissimilar-Neutral). The presentation of background contexts was modelled with a boxcar function of 12 s duration (including the presentation of the three embedded objects), and convolved with a canonical hemodynamic response function (HRF) combined with time and dispersion derivatives (Friston et al., 1998). These ‘context event’ regressors were parametrically modulated by their respective ‘context memory’ scores (i.e. the number of objects, ranging from 0 to 3, recognized as ‘old’ during the memory test). These participant-specific parametric regressors were also convolved with the HRF (and time/dispersion derivatives), allowing us to identify brain regions whose activity correlated with successful memory for embedded objects as a function of context type. 8 Object regressors were also included in the *same* GLM (corresponding to the embedded objects) as stick functions. These corresponded to the 2 × 2 factorial design (similarity, valence), with the additional incorporation of whether an object was subsequently recognized 5 days later (i.e. hit) or not (i.e. miss). Error trials (i.e. on which subjects made incorrect semantic judgments to the objects) and the presentation of both informative feedback as well the non-informative feedback (i.e. presentation of the question mark, rather than the amount of money won) were included in the GLM as regressors of no interest. The extent to which the informative feedback was correlated with any of the context or object regressors in the fMRI design never exceeded an absolute *r* of 0.25, for any individual subject. Thus, the presentation of the informative outcome is unlikely to have influenced our ability to identify neural effects that were related to the contexts or objects. Note however that regressors for button presses were *not* included in the model, as this would have eliminated our ability to examine object-related responses entirely (due to the multicollinearity of the object regressors as a whole with motor responses, given that subjects responded with a button press to every object present). While it would have been desirable to compare neural responses to the context when presented alone vs the rest of the context epoch, the lack of jitter in our experimental design meant that we were unable to separate these two responses. Additional covariates were included to capture residual artifacts related to movement (three rigid-body translations and three rotations from realignment), scanning session, heart rate and respiration. Model estimation proceeded in two stages: in the first stage, condition-specific experimental effects (parameter estimates) were obtained in a voxel-wise manner for each participant. In the second (random-effects) stage, participant-specific linear contrasts of these parameter estimates were entered into a series of ANOVAs (i.e. 2 × 2 factorial, Similarity × Valence for the context-event and context memory conditions; 2 × 2 × 2 factorial, Similarity × Valence × Recognition for the object conditions).

### 2.7 Regions of interest

We focused our analysis on the midbrain and the hippocampus, because these regions are thought to mediate the reward-related and novelty-related enhancement of episodic memory (Lisman and Grace, 2005, Ranganath and Rainer, 2003, Wittmann et al., 2005). Because all analyses were performed in group-template space (see above section regarding ANTs normalization), all anatomical search volumes had to be manually defined using the software MRIcron. Hippocampal anatomical masks (4322 voxels on the left, 4601 voxels on the right) were created by manually segmenting the hippocampus on the group T1-weighted template scan, guided by an anatomical atlas (Duvernoy, 2013). The substantia nigra/ventral tegmental area (971 voxels on the left, 979 on the right) anatomical mask was manually defined using the group magnetization-transfer-weighted template scan created using the normalization protocol employed. On MTw images, the SN/VTA can be distinguished from surrounding structures as a bright stripe (Bunzeck and Düzel, 2006, Düzel et al., 2009). Where further analysis motivated the division of the hippocampus into anterior and posterior sections, ROIs were created by segmenting the above-mentioned hippocampal image at the first coronal slice in which the uncus could be clearly observed, in line with existing recommendations in the literature (Baumann et al., 2010, Poppenk et al., 2013). Voxels anterior to and including this slice were regarded as belonging to the anterior hippocampus (1736 voxels on the left, 2046 voxels on the right), while voxels posterior to this line were regarded as being part of the posterior hippocampus.

All ROIs were defined from contrasts that were orthogonal to the contrasts of interest to allow statistical tests to be performed in an unbiased fashion. We examined the identity matrix F-contrast in each second-level model (which identifies voxels that are, on average, sensitive to the context presentations, ignoring the similarity and valence conditions) at a threshold of *p* = 0.05 uncorrected, and applied the anatomical masks to define the search volume to be used in small-volume correction. This procedure thus identifies voxels in our anatomical regions of interest that respond to the overall cohort of conditions on average (e.g. all the context-event conditions, in the context-event model). The identity matrix contrast used to create the search volume is, importantly, orthogonal to our comparisons of interest, which focus on *between*-condition differences in activation, rather than condition-specific activations relative to baseline. As such, this procedure avoids statistical double-dipping in controlling for multiple comparisons (Kriegeskorte, Simmons, Bellgowan, & Baker, 2009), and provides us a way of balancing the likelihood of Type I and Type II error without compromising statistical validity. All reported voxel-based results were initially thresholded at *p* < 0.001 uncorrected, and all reported whole-brain results were significant at a threshold of *p* < 0.05 family-wise-error corrected with small-volume correction for the particular anatomical region-of-interest in question (bilateral hippocampus or bilateral SN/VTA).

### 2.8 Hippocampal subfield delineation

To allow for anatomical delineation of the DG/CA3, we acquired an additional structural scan focused on the temporal lobes (partial volume), employing the same acquisition protocols that were used by Bonnici et al. (2012). A single-slab 3D T2-weighted turbo spin echo sequence with variable flip angles in combination with parallel imaging was employed to simultaneously achieve a high image resolution of ∼500 μm, high sampling efficiency and short scan time while maintaining a sufficient SNR. After excitation of a single axial slab the image was read out with the following parameters: resolution = 0.52 × 0.52 × 0.5 mm^3^, matrix = 384 × 328, partitions = 104, partition thickness = 0.5 mm, partition oversampling = 15.4%, field of view = 200 × 171 mm^2^, echo time (TE) = 353 ms, repetition time (TR) = 3200 ms, parallel imaging with GRAPPA × 2 in phase-encoding (PE) direction, bandwidth = 434 Hz/pixel, echo spacing = 4.98 ms, turbo factor in PE direction = 177, echo train duration = 881, averages = 1.9. For reduction of signal bias due to, e.g., spatial variation in coil sensitivity profiles, the images were normalized using a prescan and a weak intensity filter was applied as implemented by the scanner’s manufacturer. To improve the SNR of the anatomical image, four scans were acquired for each subject, co-registered and averaged. It took 12 min to obtain each scan with a total scanning time of 48 min.

After each subject’s slab was averaged, this scan was co-registered to the whole-brain structural scan (to which the functional scans were also co-registered). We then used ANTs to create a group-level hippocampal slab (using the same procedures as were employed for the whole-brain structural scan). A combined DG/CA3 mask was then manually traced on this group-level hippocampal slab by a researcher who had extensive experience with tracing hippocampal subfields. The DG/CA3 masks were limited to the anterior hippocampus because early analysis had indicated that contextual reward effects in the similar condition were specific to the anterior hippocampus. The anterior DG/CA3 masks were traced using the software developed by Hugo Kuijf (Kuijf, 2013) and based on MeVisLab (MeVis Medical Solutions AG, Bremen, Germany), separately for each hemisphere, with the first coronal slice where the uncus can be clearly seen counted as the first slice belonging to the anterior hippocampus. The masks were segmented according to the recently published protocol of (Wisse et al., 2012), and based on experience from segmenting high-resolution data, using detailed landmarks described in the Atlas of the Human Brain were as additional guideline. Based on Wisse et al. (2012) the delineation of CA3 starts 1.4 mm anterior to the point where the uncus separates from the hippocampus on coronal images. In the atlas of the human brain there is also almost no CA3 in the anterior hippocampal head (Mai, Paxinos, & Voss, 2008). Therefore, we began segmenting the CA3 two slices anterior to the point where the uncus separates from the hippocampus. The border between the CA1 and CA3 subfield was formed by the lateral-most point of DG, by drawing a vertical line to the superior border of the hippocampus. We were not able to trace CA2, which was therefore counted towards CA3.

### 2.9 Psycho-physiological models

Psycho-physiological (PPI) models were employed to examine trial-by-trial functional coupling of regions of interest in each of the different context conditions. Such analyses allow one to show that activity in a distant region can be accounted for by an interaction between the influence of a source region and an experimental parameter (Friston et al., 1997). We used a PPI analysis to examine if the right SN/VTA (our source region, derived from observation of results peak coordinates in second-level contrasts; see Section 3 for further detail) significantly influenced activity in the bilateral anterior hippocampus in relation to memory in each of the context conditions. SPM was used to extract the time series from a 2 mm sphere in the SN/VTA (location derived from the simple-effects contrasts that were performed as a follow up from the interaction analysis in the whole-brain voxel-based analysis). Five separate PPIs were run (one for each context condition compared to baseline, and one directly comparing the similar-reward with the similar-neutral), and parameter estimates from the bilateral anterior hippocampus (see Section 3 for motivation) were then extracted from all PPI models, and subjected to correlational analysis with the memory scores *d*′ and RT measures from the encoding-stage task.

## 3 Results

### 3.1 Contextual reward improves memory for embedded objects *selectively* in the similar condition

**Fig. 2:**
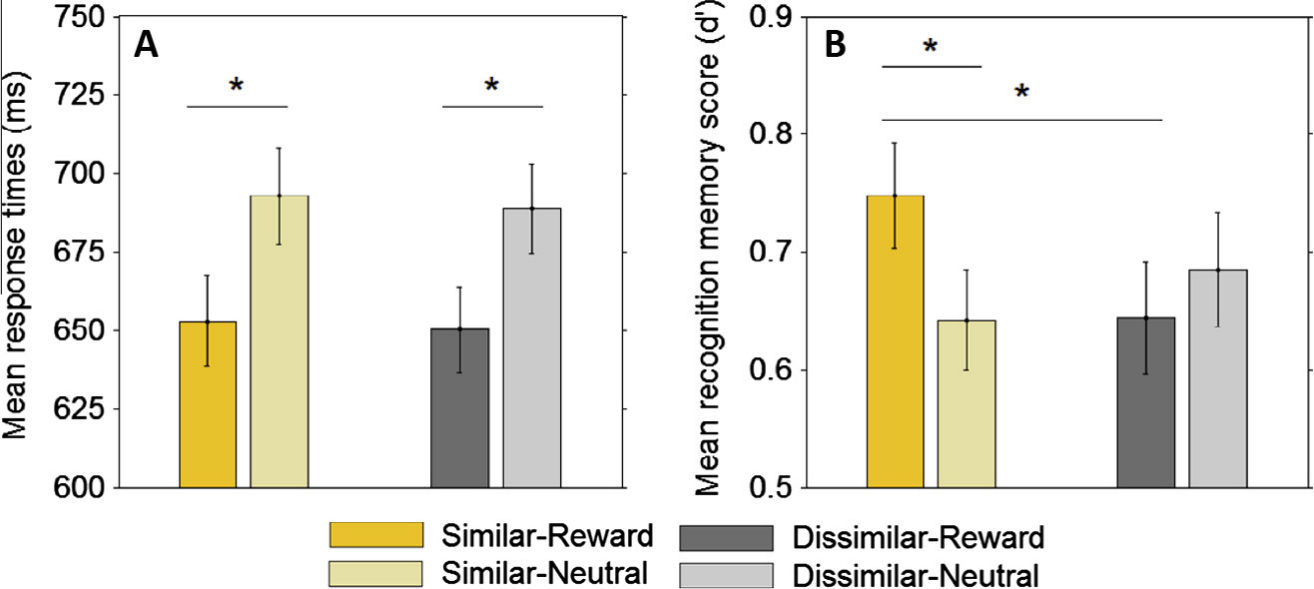
Behavioural performance on encoding-stage task and recognition memory. Participants were quicker to respond to objects when there was a rewarding context in the background (A), in both the similar and dissimilar context conditions. Despite successful and comparable context conditioning in the similar and dissimilar conditions, recognition memory (indexed by *d*′) measured after a five day delay was enhanced by reward in the similar context condition, but not the dissimilar (B). Error bars are ±1 SE.

After context conditioning, all subjects verbally reported which contexts were rewarded and which were not with full accuracy. RTs from the conditioning stage indicated successful reward conditioning that was comparable in the similar and dissimilar condition (assessed with a 2 × 2 similarity × valence ANOVA, main effect of valence: *F*(1, 24) = 5.30, *p* < 0.03; *p* > 0.4 for the similarity × valence interaction and main effect of similarity; mean RT speeding of 20.65 and 25.59 ms, SD of 51.05 and 53.80 in the similar and dissimilar condition, respectively). Accuracy on the object semantic-judgment task performed during the scanning session was very high (mean accuracy = 96.20%, SD = 0.03), and was not affected by context similarity or valence (assessed with a 2 × 2 similarity × valence ANOVA; all main effects and interaction *p* > 0.2). Participants made faster responses to objects when a rewarding background context was present (valence effect, *F*(1, 24) = 30.38, *p* < .001; Fig. 2A), and this reward effect was not modulated by context similarity (similarity × valence interaction and main effect of similarity both *p* > .0.3), indicating successful and comparable reward-conditioning in both the similar and dissimilar context pairs.

Overall, participants showed above chance memory for the objects during a recognition memory test five days after encoding (mean *d*′ = 0.67, SD = 0.19). Participants were, however, more likely to recognize an object if it had been presented with a similar-rewarded context, compared to a similar-neutral or a dissimilar-rewarded context (Fig. 2B; similarity × valence interaction, *F*(1, 24) = 4.64, *p* = 0.042; similar-rewarded versus similar-neutral, *t*(24) = 2.68, *p* = 0.01; similar-rewarded versus dissimilar-reward, *t*(24) = 2.27, *p* = 0.03; similar-rewarded versus dissimilar-neutral, *p* > 0.1). No main effect of context similarity or valence was found on object recognition (both *p* > 0.2), and post hoc *t*-tests found no valence effect in the dissimilar context condition (dissimilar-rewarded versus dissimilar-neutral, *p* > 0.4). No main effects or interaction of context similarity and valence were observed in remember rates (all *p* > 0.2; Supplementary Fig. 1) or know rates (all *p* > 0.098; Supplementary Fig. 1). Rates of *confident* recognition mirrored the pattern of effects shown in *d*′ (similarity × valence interaction, *F*(1, 24) = 6.91, *p* = 0.015; both main effects of similarity and valence *p* > 0.5; Supplementary Fig. 1), albeit less strongly (similar-rewarded versus similar-neutral, *t*(24) = 1.85, *p* = 0.078; similar-rewarded versus dissimilar-reward, *p* > 0.03; similar-rewarded versus dissimilar-neutral, *p* > 0.7). Rates of *unsure* recognition showed no main effects or interaction between similarity and valence, however (all *p* > 0.1). To verify that the observed effects on *d*′ were not modulated by the presentation of the outcome (which was omitted on 50% of trials, to enable us to de-correlate the outcome presentation from the presentation of the context stimuli, in the fMRI design; see Section 2 for more detail), we split trials according to whether the outcome was shown or not on that trial, and analyzed the *d*′ scores with a 2 × 2 × 3 (similarity × valence × outcome presentation) ANOVA. The three-way interaction in this analysis was not significant (*p* > 0.3), and no main effect of outcome presentation or interactions between outcome presentation and similarity or valence were observed (all *p* > 0.3). This indicates that the observed effects of similarity and valence on *d*′ memory scores were unlikely to be merely a result of the presentation of informative outcomes regarding trial-wise winnings.

These results indicate that a rewarding context affords a mnemonic benefit *selectively* in the similar condition. Notably, the observed asymmetry in the recognition effects between the similar and dissimilar condition are unlikely to be due to differences in context reward conditioning, working memory load associated with context discrimination or other attentional differences, as subjects were well conditioned prior to the encoding stage, had 4s on each encoding trial to examine the context picture alone before the objects were presented, and demonstrated no differences in context conditioning as indexed by encoding-stage response times (RTs; no similarity × valence interaction in RTs, *p* > 0.3; Fig. 2A). These behavioural results indicate that contextual reward enhances memory for embedded neutral events particularly when context discrimination poses demands on neural processes that depend on the hippocampus (in our case, on pattern separation). We therefore examined whether the observed benefits in recognition memory in the similar-reward condition would be underpinned by activation of the hippocampus (DG/CA3 subfield in particular) together with a reward-related recruitment of the SN/VTA.

### 3.2 Context-related activation of the anterior DG/CA3 and SN/VTA tracks successful memory formation in the similar-reward condition

**Fig. 3:**
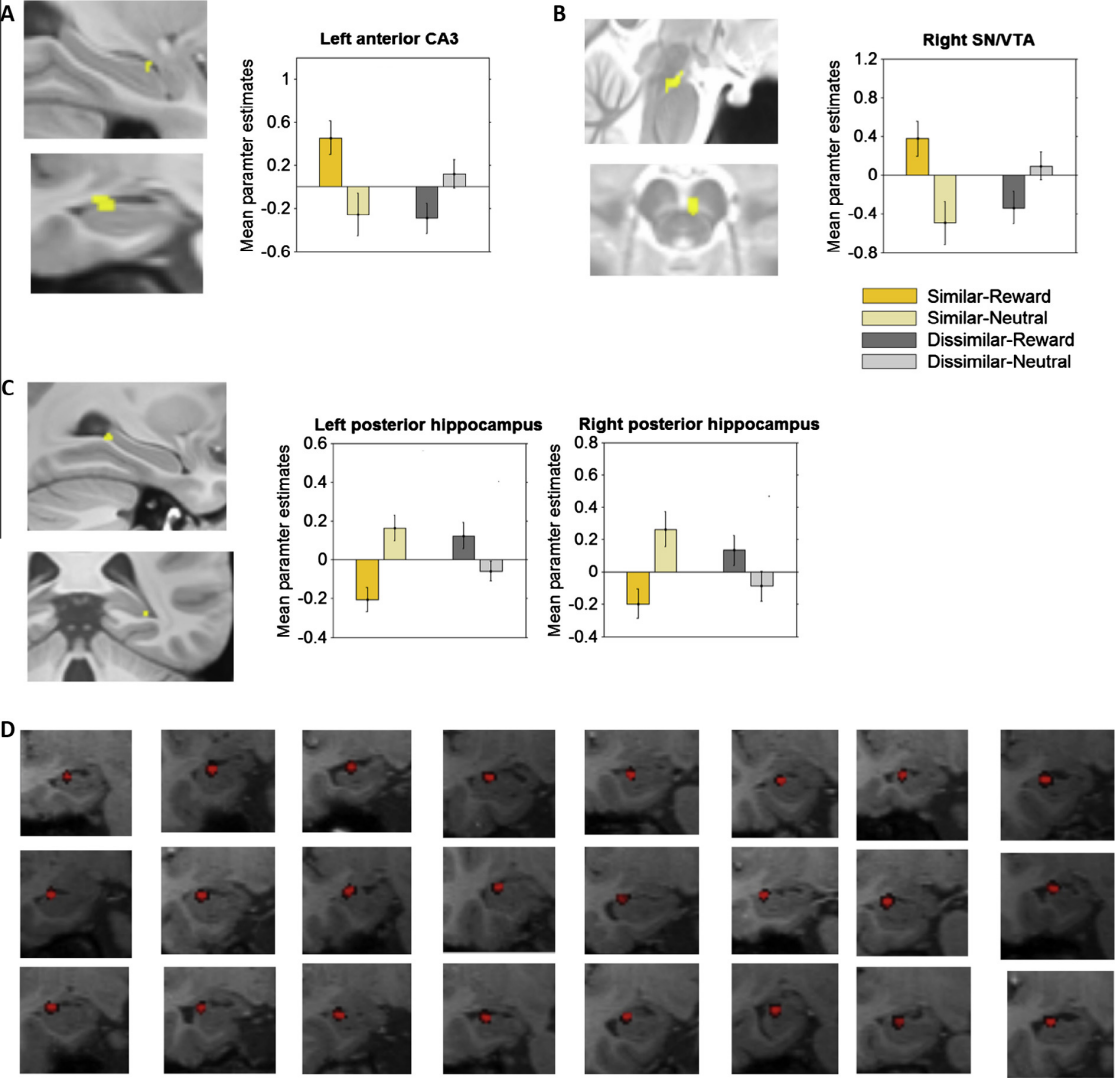
The hippocampus and SN/VTA track memory. Activation of the left anterior hippocampal CA3 (A) and right SN/VTA (B) in response to the contexts were found to track object memory more in the similar-reward condition compared to the similar-neutral or dissimilar-reward. In contrast, activation of the bilateral posterior hippocampus was found to track memory for objects encountered in the similar-neutral context (C; right hippocampal cluster pictured). Inverse transformations of the DG/CA3 ROI from the group template space to the native space of each individual participant confirmed that this hippocampal cluster mapped onto the DG/CA3 region of the hippocampus in every single participant (D). All error bars are ±1 SE.

We employed a single first-level fMRI model that included regressors describing each 12 s context epoch (by similarity and valence), each object presentation (by context similarity, context valence, and object-recognition success), and ‘context-memory’ parametric modulators (parametric modulators applied to the context epoch regressors, describing the number of co-presented objects out of three that were later successfully recognized; see Section 2 for more detail). Examination of these subject-specific context-memory regressors allowed us to identify neural responses that varied as a function of context-related memory in each of the four context conditions, after controlling for object-related (rather than context-related) responses. The first-level context-memory contrasts were included in a second-level 2 × 2 ANOVA (Similarity: similar, dissimilar; Valence: reward, neutral), and examination of the similarity by valence interaction at a significance threshold of *p* < 0.05 FWE (with small-volume correction for the bilateral hippocampus and SN/VTA search volumes; see Section 2 for more detail) revealed clusters in the left anterior DG/CA3 subfield of the hippocampus, the right SN/VTA, and the bilateral posterior hippocampus (Fig. 3A–C). Further examination of the constituent positive and negative interaction contrasts revealed two distinct networks of activity across the different context conditions. The positive interaction contrast revealed activation clusters in the left anterior hippocampal DG/CA3 subfield and right SN/VTA (Fig. 3A and B; Left DG/CA3: FWE *p* = 0.030, *t*(23) = 3.60, *Z* = 3.47, peak coordinates = −27.4, 5.5, 9.3, 28 voxels; Right SN/VTA: FWE *p* = 0.018, *t*(23) = 3.40, peak coordinates = 2.6, −4.5, 4.2, 45 voxels). In contrast, the negative interaction revealed significant clusters in the posterior bilateral hippocampus (Fig. 3C). No other significant activation was observed in the main effect of similarity, or main effect of valence contrasts. The anterior hippocampal cluster identified in the positive interaction contrast did appear to be localized to the DG/CA3 in anatomical group space. To verify that the specialized localization protocols employed were sufficiently accurate as to allow for such fine localization, we used the inverse mapping tools from the normalization protocols (Advanced Normalization Tools; see Section 2 for more detail) to verify that the group-level hippocampal cluster reported here did indeed map onto voxels from the DG/CA3 hippocampal subfield in each participants’ native space. Results from this inverse mapping indicate that the group-level-significant cluster in the anterior hippocampus did indeed correspond to voxels from the DG/CA3 subregion in every single participant (Fig. 3D).

To determine which simple effects were driving the positive interaction in the DG/CA3 and SN/VTA, we examined each simple-effects voxel-based contrast that made up the positive interaction, looking specifically for activation in these same functional ROIs (Fig. 3). We decided to examine the simple effects using the voxel-based contrasts (rather than by extracting parameter estimates from each ROI and conducting *t*-tests for each simple effect at the beta level) so as to maintain a consistent whole-brain significance threshold throughout this analysis. Examination of the simple-effects contrasts indicated that the interaction effects in the anterior DG/CA3 and SN/VTA were driven mainly by greater activations in these regions in the similar-reward context compared to the similar-neutral. Of the four simple-effects contrasts (comparing similar-reward vs similar-neutral, dissimilar-neutral vs dissimilar-reward, similar-reward vs dissimilar-reward and dissimilar-neutral vs similar-neutral), only the similar-reward > similar-neutral contrast found any significant voxels (even at the relatively lenient threshold of *p* = 0.001 uncorrected) in these functional ROIs. These results are in line with the hypothesis that recruitment of hippocampus and SN/VTA mediate the selective memory enhancement observed in the similar-reward condition.

Context and object-related regressors describing memory were allowed to compete for variance in the *same* fMRI general linear model (see Section 2 for more detail). This general linear model reveals significant activations relating to variance that is *uniquely* explained by each regressor, and thus allows us to examine context-related activation that is not contaminated by object-related responses. Our ability to control for such object-related responses in our fMRI analysis allows us to infer that the observed context-related memory effects were unlikely to have been a mere product of summated object-related responses. Examining the *object*-related regressors using the voxel-based approach found no significant effects across the entire partial volume in support of successful memory, either as a function of context similarity, context valence, or an interaction between these two factors. Activation relating to the presentation of the objects themselves (independent of memory, i.e. object > baseline) did however identify clusters in the lateral prefrontal cortex, insula, parahippocampal cortex, perirhinal cortex and cerebellum (Supplementary Fig. 2B; note the limited coverage of the partial volume, Supplementary Fig. 2A). This object-related activation relates both to the presentation of objects as we as to subsequent motor responses, since button-presses were not included in the first-level GLM.

Our experimental design does not enable us to rule out a role for object-related neural activations in support of memory, since sub-threshold activation or activations that are shared by context and object regressors (which do not appear as results from the GLM) may additionally contribute to the observed behavioural effects. Given that shared variance is discarded in the reported model, however, the inclusion of both context- and object-related regressors in the *same* fMRI model may have impaired our ability to identify object-related effects. To further confirm that the context-related effects in the DG/CA3 and SN/VTA were not a summation of object-related responses, therefore, we constructed an additional fMRI model that did *not* require object-related responses to compete with context-related responses, and used this model to verify that the observed context-related effects (i.e. shown in Fig. 3A and B) were not object-related in origin (see Supplementary Results for more detail).

In addition to the results relating to improved memory in the similar-reward condition, the negative similarity × valence interaction contrast also revealed significant clusters in the bilateral posterior hippocampus (right posterior hippocampus pictured in Fig. 3C; Right: FWE *p* = 0.025, *t*(23) = 3.70, *Z* = 3.52, peak coordinates = 27.6, −22.0, 16.1, 21 voxels; Left: FWE *p* = 0.010, *t*(23) = 3.90, peak coordinates = −31.4, −18.0, 18.2, 164 voxels). Simple effects comparisons revealed that these activations were driven mainly by differences in the similar condition, but in the opposite direction to the results reported so far, tracking memory in the similar-neutral condition more than in the similar-reward condition (Fig. 3C). Overall, these results are suggestive of a functional dissociation in the processing of rewarding and neutral contexts, with rewarding contexts modulating memory for embedded stimuli via the anterior hippocampus, and neutral contexts modulating memory (without necessarily producing better subsequent recognition) via the posterior hippocampus.

### 3.3 Successfully conditioned contexts elicit SN/VTA activity in the similar-reward condition

**Fig. 4:**
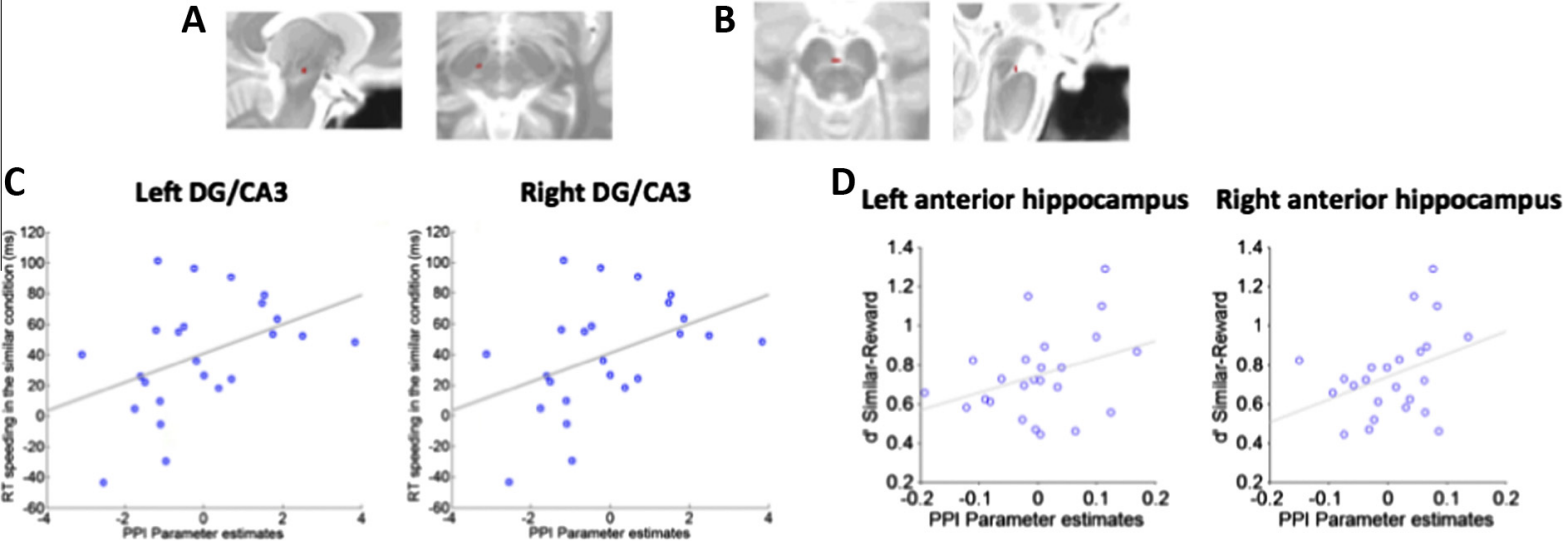
SN/VTA responding and connectivity in support of memory and context conditioning in the similar condition. The left SN/VTA (A) responded more strongly to the similar-reward context as compared to the similar-neutral, whereas the mid SN/VTA (B) responded more strongly to the similar-reward context as compared to the dissimilar-reward. In the similar condition, coupling between the right SN/VTA and bilateral DG/CA3 was correlated, across all subjects, with the amount of RT speeding in the similar condition (D), while coupling between the right SN/VTA and the anterior hippocampus was correlated with individual differences in memory (in the similar-reward condition; D).

Using the same first-level fMRI model, we were also able to identify activations relating to processing of the similar and dissimilar contexts themselves. We examined these context-related activations to identify brain regions that support specific reward learning in the similar condition (i.e. in the face of perceptual similarity), and that might reveal additional asymmetries in the processing of the similar and dissimilar contexts that might explain the striking behavioural pattern of memory effects. First-level contrasts relating to the 12 s context epochs were entered into a second-level 2 × 2 (Similarity × Valence) ANOVA. Surprisingly, neither the main effect or interaction contrasts revealed any significant voxels across the entire partial volume. The lack of a main effect of valence in the SN/VTA was unexpected, and motivated us to conduct further exploratory analysis. We directly contrasted the similar-reward with the similar-neutral condition, and the dissimilar-reward with the dissimilar-neutral condition, in order to further examine the reward-related response in each condition separately. While direct comparison of the similar-reward compared to the similar-neutral context condition revealed a cluster in left SN/VTA (Fig. 4A; peak FWE *p* = 0.041, *t* = 3.31, 6 voxel cluster), comparing the dissimilar-reward with the dissimilar-neutral condition found no surviving voxels in the SN/VTA, even at the very lenient threshold of *p* < 0.05 uncorrected. While these negative results relating to processing of the rewarding context in the dissimilar condition do not allow us to infer that the SN/VTA response to the dissimilar-rewarding context was entirely absent, the overall pattern of results were suggestive of a difference in the reward-related SN/VTA response in the similar and dissimilar conditions. To further explore this possibility, we directly compared the similar-reward > dissimilar-reward contexts, and this comparison revealed a cluster in the middle of the SN/VTA (Fig. 4B; peak FWE *p* = 0.033, *t* = 3.53, 11 voxels). Examination of the reverse contrast (dissimilar-reward > similar-reward) revealed no significant voxels in the SN/VTA, even at the very lenient threshold of *p* = 0.05 uncorrected. These results indicate that the reward-related response of the SN/VTA was stronger in the similar compared to the dissimilar condition, despite comparable context-reward learning in the similar and dissimilar conditions (as indexed by RT speeding; Fig. 2A; see Supplementary results for further evidence that links the strength of the SN/VTA response successful context conditioning). Overall, these findings suggest a selectivity in the SN/VTA response to the similar-reward context. This selectivity of the SN/VTA response may provide a potential mechanism that allows specific reward associations to be formed with individual contexts, without generalizing to perceptually similar but motivationally neutral contexts. Further, the asymmetry of the SN/VTA response in the similar and dissimilar conditions, found using exploratory analyses, suggests that the underlying discriminatory processing circuits may influence the extent to which context representations are able to drive robust reward-related responding in the SN/VTA. This difference in the SN/VTA response in the similar and dissimilar conditions may additionally explain the lack of a memory benefit in the dissimilar-reward as compared to the similar-reward condition. Interestingly, we did not observe increased hippocampal activity in the similar-reward condition. Indeed, no effects were observed in the hippocampus in relation to the context-event regressors for all reported contrasts, even at the threshold of *p* < 0.001 uncorrected.

### 3.4 Connectivity between the right SN/VTA and the anterior hippocampus is correlated with reward-related RT speeding and successful memory encoding in the similar-reward condition

The findings presented so far implicate the anterior and posterior hippocampus respectively in the modulation of memory by the similar-reward and similar-neutral contexts. To further examine functional connectivity (rather than co-activation) between the anterior DG/CA3 and the SN/VTA, we conducted a psycho-physiological interaction (PPI) analysis to see if connectivity between these regions was linked to the reward-related effects observed in our task. We seeded a PPI using the peak coordinate derived from the activation cluster in the right SN/VTA (identified using the similar-reward > similar-neutral contrast from the context-memory analysis), and extracted parameter estimates from the positive PPI contrast for the bilateral DG/CA3 hippocampus (anatomically defined; see Section 2 for more detail). In a between-subjects analysis, parameter estimates from these regions were then subjected to correlational analysis with *d*′ memory scores and with the RT measures of context conditioning.

Across all subjects, functional coupling between the SN/VTA and the bilateral anterior DG/CA3 was linked to successful reward conditioning (indexed by reward-related RT speeding; Fig. 2A) in the similar condition. Greater coupling between the right SN/VTA and bilateral anterior DG/CA3 in the similar-reward condition was correlated with greater reward-related RT speeding in the similar condition (Fig. 4C; left: *r* = 0.43, *p* = 0.018 one-tailed; right: *r* = 0.43, *p* = 0.018 one-tailed). No such correlation between reward-related RT speeding and DG/CA3-SN/VTA coupling was observed in the dissimilar condition (*p* > 0.3 for left and right).

No between-subject relationships were seen between the amount of SN/VTA-DG/CA3 coupling and the observed memory effects (*p* > 0.1; note that reward-related RT speeding and memory in the similar-reward condition are *not* correlated across all subjects, *p* > 0.3). We repeated this analysis to examine if individual differences in memory were linked to the amount of SN/VTA coupling with the *anterior* hippocampus as a whole (anatomically defined; see Section 2 for more detail), since the previous analysis had indicated that the contextual effects may have been differentially localized to the anterior and posterior hippocampus in the similar condition. This exploratory analysis found that recognition memory in the similar-reward condition was correlated (across all subjects) with the coupling between the SN/VTA and the anterior hippocampus (Fig. 4D; left: *r* = 0.35, *p* = 0.046 one-tailed; right: *r* = 0.36, *p* = 0.040 one-tailed). Increased coupling between the SN/VTA and the *anterior* hippocampus during encoding (comparing the similar-reward with the similar-neutral) was therefore associated with better subsequent memory in the similar-rewarded condition.

These results indicate that functional connectivity between the anterior hippocampus and the SN/VTA may underlie successful context conditioning in addition to the observed memory effects in the similar-reward condition. Interestingly, while this was specific to the anterior DG/CA3 sub-region in the case of context learning, no such specificity was observed in the case of contextual memory modulation: while individual differences in reward-related RT speeding were linked to SN/VTA coupling with the anterior DG/CA3, the extent to which subjects’ memory was improved by contextual reward (in the similar condition) was linked to SN/VTA coupling with the anterior hippocampus as a whole. These results point towards a role for the right and left anterior hippocampus in supporting memory enhancement in the similar-reward condition, and further support the hypothesis that interactions between the SN/VTA and the hippocampus underlie the memory benefit in the similar-reward condition. Additionally, these results further indicate that successful reward learning in the similar condition is supported by interactions between the DG/CA3 sub-region of the hippocampus and the SN/VTA.

## 4 Discussion

By varying the similarity of our context stimuli, we had set out to vary the extent to which context discrimination should theoretically depend on the hippocampus (and the DG/CA3 region in particular). Lesion data from humans and animals have demonstrated that an intact hippocampus is necessary for reliable disambiguation of perceptually similar scenes, with hippocampal damage leading to deficits in the ability to reliably distinguish perceptual similar stimuli (Graham et al., 2006, Hannula et al., 2006, Lee et al., 2005, McHugh et al., 2007, Neunuebel and Knierim, 2014, Watson et al., 2013). Furthermore, functional imaging studies have related the CA3 region of the hippocampus to distinct representations of perceptually similar stimuli (Bakker et al., 2008, Bonnici et al., 2012), even for stimuli that have been made familiar via repeated exposure (Berron, Schutze, Maass, Kumaran, & Duzel, 2013). Therefore, it seems plausible that the specific SN/VTA response to the similar rewarding context in our experiment (i.e. that did not generalize to the similar neutral context) was related to hippocampal disambiguation of context representations. Indeed, the degree of behavioural context conditioning in the similar condition (as indexed by the speeding of responses on trials with rewarding contexts) was correlated with functional connectivity between the SN/VTA and the anterior DG/CA3 (Fig. 4C). Such a relationship was absent in the dissimilar context condition. This neural difference between the similar and dissimilar context occurred despite comparable reward conditioning of the context stimuli, as indexed by reward-related RT speeding in both the conditioning and encoding stages of the experiment.

The idea that disambiguation of similar scenes should rely on hippocampal representations is in line with recent assertions that the hippocampus is part of a representational system that spans perceptual and memory functions (Lee et al., 2012, Mundy et al., 2013, Nadel and Peterson, 2013). While different researchers have emphasized the importance of the hippocampus for binding (see Yonelinas, 2013 for recent review), relational memory (Eichenbaum and Cohen, 2001, Konkel, 2009), or the construction of coherent spatial representations specifically (Maguire & Mullally, 2013), these theories (and their associated evidence) commonly point towards the hippocampus as being crucial for perceptual functions, in addition to memory. Within this context, the pattern separation abilities of the hippocampus refer not just to the need for incoming representations to be stored separately from existing memories (even in the face of perceptual overlap), but also the need for concurrently perceived overlapping stimuli to be represented separately in the brain (Nadel & Peterson, 2013). In support of the idea that the hippocampus plays a role in disambiguation at a perceptual or representational level, several experiments indicate that the hippocampus remains involved in maintaining disambiguated representations of similar scenes not just at first encounter (i.e. in relation to encoding or the creation of novel mnemonic representations), but on an ongoing basis (i.e. even as the scenes get increasingly familiar). Scene stimuli have been shown to be represented distinctly in the CA3 subfield specifically (but not in other regions, e.g. hippocampal CA1) even when subjects have been extensively familiarized with the stimuli (Berron et al., 2013), and hippocampal-lesioned patients fail to show any improvements in their ability to disambiguate scenes with overlapping features (which would be indicative of a switch to hippocampal-independent discrimination strategies), even with repeated exposures and direct trial-wise feedback regarding their disambiguation accuracy (Lee et al., 2005). Similarly, depleting hippocampal neurogenesis in mice produces a deficit in the ability to disambiguate similar contexts that cannot be compensated for with extensive training (Tronel et al., 2012). Given such findings, it seems likely that the ongoing disambiguation of the similar scenes in our experiment would have relied on orthogonal context representations in the hippocampus, and that learning about the similar-reward context in particular would have relied on these representations driving the reward-related response in the SN/VTA. In support of this idea, we did observe that, across all subjects, coupling between the SN/VTA and the anterior DG/CA3 was linked to reward-related RT speeding in the similar condition alone (Fig. 4C). While one might have expected to find a main effect of similarity in the similar compared to the dissimilar context condition (in the within-subjects univariate analysis), we did not find greater hippocampal activation when subjects were faced with the similar compared to dissimilar context pictures. It is worth noting, however, that such distinct univariate effects are often not found in fMRI studies that aim to examine hippocampal pattern separation. Such studies have generally not reported such results (i.e. greater hippocampal activity when facing ambiguous compared to unambiguous stimuli) in the hippocampus, despite such a univariate contrast (i.e. comparing exposure to similar vs dissimilar stimuli) being the most straightforward of fMRI analysis approaches. Instead, these studies have employed repetition suppression paradigms (Azab et al., 2013, Bakker et al., 2008, Lacy et al., 2011) or MVPA (Berron et al., 2013, Bonnici et al., 2012, Huffman and Stark, 2014), which examine hippocampal representations rather than focusing on overall levels of activation in the hippocampus per se.

Successful learning about the rewarding context in the similar condition was associated with responding in the SN/VTA that did not generalize to the similar-neutral context. Context-related activation of the SN/VTA was greater in the similar-reward compared to the similar-neutral condition; while significant SN/VTA activation was noted comparing the similar-reward > similar-neutral context regressors that were independent of memory (Fig. 4B), this greater SN/VTA response in the similar-reward condition was also tightly linked to the context-related memory scores, and thus appeared more robustly in our analysis of the context-memory regressors (Fig. 3B). This specific SN/VTA response to the similar-rewarded contexts is highly relevant in view of physiological evidence that the hippocampus can disinhibit dopaminergic neurons via two polysynaptic pathways. One pathway originates in the subiculum and relays by the nucleus accumbens and the ventral pallidum (Floresco et al., 2001, Floresco et al., 2003, Grace et al., 2007, Legault and Wise, 1999, Lodge and Grace, 2006), while the other originates at CA3 and relays at the caudo-dorsal lateral septum (Luo et al., 2011). The ability of the hippocampus to disinhibit the SN/VTA via such pathways (see Lisman & Grace, 2005, for review) may potentially account for our observation (found using exploratory analysis) that SN/VTA activity in the similar-reward condition was stronger than any reward-related response in the dissimilar condition (Fig. 4B).

The difference in the reward-related SN/VTA response between the similar and dissimilar conditions was accompanied by differences in the effects of contextual reward on memory. Contextual reward improved memory for embedded objects only in the similar condition, i.e. recognition memory for objects encountered in the similar-reward context was higher compared to memory for objects in the similar-neutral context (Fig. 2B). Within-subject fMRI analysis based on subsequent memory performance showed that this selective memory enhancement was related to recruitment of the anterior hippocampus and SN/VTA during the entire context epoch (Fig. 3A and B). Our specialized anatomical normalization protocol (optimized for hippocampal subfields and the SN/VTA) allowed us to localize this memory-related hippocampal activation specifically to the subfield DG/CA3 (Fig. 3A; consistently localized to DG/CA3 in each participants native space, Fig. 3D). This selectivity and specificity of activation in the DG/CA3 and SN/VTA is remarkable because it is fully consistent with the aforementioned, newly discovered pathway from CA3 to SN/VTA (Luo et al., 2011), and was observed alongside PPI findings that linked SN/VTA-DG/CA3 coupling to individual differences in the reward-related RT speeding in the similar condition. Consistent with our findings, a previous study from Wolosin, Zeithamova, and Preston (2012) found that, when subjects were rewarded for intentionally remembering object pairs, reward-related changes in the DG/CA2–3 and SN/VTA were related to successful memory performance. The data reported here extend the importance of the DG/CA3 and SN/VTA to *contextual* reward effects on object memory, and further demonstrate that the extent to which hippocampal computations are necessary for reward discrimination can influence the reward-related memory benefits that are observed, by modulating the strength of the reward-related SN/VTA response.

While coupling between the DG/CA3 and the SN/VTA did not show any *between*-subject statistical relationship with memory, the amount of functional coupling between the SN/VTA and the bilateral anterior hippocampus was correlated with memory in the similar rewarded context (across all subjects; Fig. 4D). While these findings were found using exploratory analysis, they do suggest that interaction between the hippocampus and SN/VTA may have been involved in producing the observed memory effects. Hippocampal outputs triggered by the processing of a rewarding context could have caused tonic disinhibition of the SN/VTA (Blaha et al., 1997, Brudzynski and Gibson, 1997, Floresco et al., 2001, Floresco et al., 2003, Legault and Wise, 1999, Lodge and Grace, 2006) thereby increasing the likelihood that embedded events will trigger phasic SN/VTA activation despite being non-predictive of reinforcement. Alternatively, or additionally, dopamine release triggered by a reward-predicting context might have increased the availability of plasticity-related proteins in the hippocampus, which, in turn, would stabilize memory representations for embedded neutral objects (a phenomenon known as synaptic tagging and capture; Bethus et al., 2010, Chowdhury et al., 2012, Frey and Morris, 1997, McNamara et al., 2014, O’Carroll et al., 2006, Sajikumar and Frey, 2004); see Redondo & Morris, 2011, for review). While the psychological conditions that lead to SN/VTA activation (and presumed dopamine release) differ, the mechanisms that underlie improved memory in our study are likely to be similar to the mechanisms that have been noted to underlie reward-related memory improvements in previous work (Adcock et al., 2006, Wittmann et al., 2005). Experimental work has begun to outline molecular mechanisms that may determine the selectivity of cross-stimulus memory enhancements in the synaptic tagging and capture framework (see Alarcon, 2015, for detailed review), and specificity in the behavioural phenomenon has additionally been noted in the literature (Lisman et al., 2011). While our understanding of these detailed underlying mechanisms is still incomplete, these considerations may play an important role in determining the extent to which rewards are allowed to improve memory for non-reward-predicting stimuli that temporally co-occur. For instance, if a long-lasting up-regulation of plasticity-related proteins was restricted to post-synaptic compartments (rather than being up-regulated in entire neurons), the memory penumbra could be restricted to temporally proximal stimuli (as in our study) because their representations could be more likely to converge on the same post-synaptic compartments.

Surprisingly, subsequent memory was not improved by contextual reward in the dissimilar condition (Fig. 2B). Cross-enhancement of object memory by the rewarding contexts was only observed in the similar condition, a finding that cannot be explained by differences in conditioning between the similar and dissimilar conditions, since context conditioning in the similar and dissimilar conditions were comparable in both the conditioning and encoding stages of the experiment (i.e. reward-related RT-speeding did not significantly differ in the similar vs dissimilar condition; Fig. 2A). Given the lack of a similarity effect on the extent of reward-related RT speeding, as well as the lack of a relationship (across all subjects) between RT speeding and the memory benefit in the similar-reward condition (*p* > 0.3), it also seems unlikely that the observed pattern of memory effects may have come about due to differences in attentional engagement in the similar vs dissimilar condition. Instead, the overall pattern of behaviour (improved object memory by contextual reward in the similar but not the dissimilar condition) and the observed neural effects suggest that the strength of the reward-related response in the SN/VTA may ultimately determine the extent to which memory is subject to cross-enhancement from reward. Indeed, existing evidence for cross-enhancement of incidental memory performance is mixed: previous tests in humans that have used appropriate delays between encoding and memory test (roughly 4–6 h; i.e. to target memory persistence rather than improved immediate recall due to attentional factors) have found mixed results for such cross-enhancement of memory by unrelated reward-predictive cues (Gruber et al., 2014, Murayama and Kitagami, 2014, Murty and Adcock, 2014, Wittmann et al., 2011). These studies do additionally contain clues about the reason for such discrepancy in the literature, however: in these previous studies, inter-trial or individual differences in the strength of the SN/VTA response were found to be linked to subsequent memory effects (Gruber et al., 2014), or the hippocampus’ sensitivity to the events to be remembered (Murty & Adcock, 2014). As such, natural fluctuations in the strength of the SN/VTA response may serve to determine whether such cross-stimulus memory effects are allowed to emerge. The findings reported here are consistent with this idea: exploratory analysis indicated that the SN/VTA response in the similar condition was stronger than the reward-related response in the dissimilar condition, which mirrors the pattern of memory effects observed. In addition to providing further support for the idea that the response of the SN/VTA may determine such cross-stimulus memory effects, our results additionally indicate that the neural circuits involved in disambiguation may have a systematic effect on the strength of the SN/VTA response, with predictable effects on subsequent memory. On the basis of our data, one might thus expect that other conditions that involve hippocampal drive of the SN/VTA may additionally be effective in producing cross-stimulus memory modulation (i.e. whether they invoke pattern separation processes or not).

While the anterior hippocampus tracked memory for objects that were presented in the similar-rewarded context (the focus of the discussion thus far), *posterior* hippocampal regions were more involved in tracking memory in the similar-neutral context. Such a segregation along the long hippocampal axis, with reward and affective functions linked to the anterior hippocampus and “cold” cognitive functions to the posterior hippocampus has been predicted previously (Fanselow and Dong, 2010, Poppenk et al., 2013), but, to our knowledge, has not been previously demonstrated in the recognition memory literature in humans. The results presented here indicate that context representations may be organized in the hippocampus in an analogous way, i.e. according to the affective qualities associated with the context at hand.

Our results show a surprisingly tight connection between the neural circuitry recruited in the processing of a rewarding context and the influence that such a context exerts on memory for the events that are embedded within it. A mnemonic enhancement of embedded events is only evident when pattern separation is required to maintain the integrity of the context’s value associations, and the findings presented here indicate that the reason for this link lies in the recruitment of the hippocampal–SN/VTA loop when pattern separation demands for context discrimination are high. These findings are compatible with the existence of a pathway linking subfield CA3, where pattern separated representations of environments are likely to be maintained, to the SN/VTA (Luo et al., 2011). Thus, we have identified a role for the functional loop between the anterior hippocampus and SN/VTA in maintaining undistorted representations of environmental value and in modulating long-term memory for embedded events.

## Author contributions

E.L., D.K., R.D. and E.D. designed research; E.L. performed the research and analyzed data; D.B. and R.K. provided analytic assistance; E.L., D.K., E.D. and R.D. wrote the paper.

The authors declare no competing financial interests.
