# Supplementary material for: Context-specific activation of hippocampus and SN/VTA by reward is related to enhanced long-term memory for embedded objects
Source: Neurobiol Learn Mem. 2016 Oct;134(Pt A):65–77. doi: 10.1016/j.nlm.2015.11.018 (PMC5045461; doi:10.1016/j.nlm.2015.11.018)
Supplement: Supplementary data 1 [file mmc1.pdf]

Supplementary materials

Supplementary Figure 1: Memory performance. Mean rates of remember, know, sure recognition and unsure (guess) recognition. All rates are corrected for false alarms during memory test. No significant main effects or interactions were found in the analysis of any of these measures (all $p>0.1$).

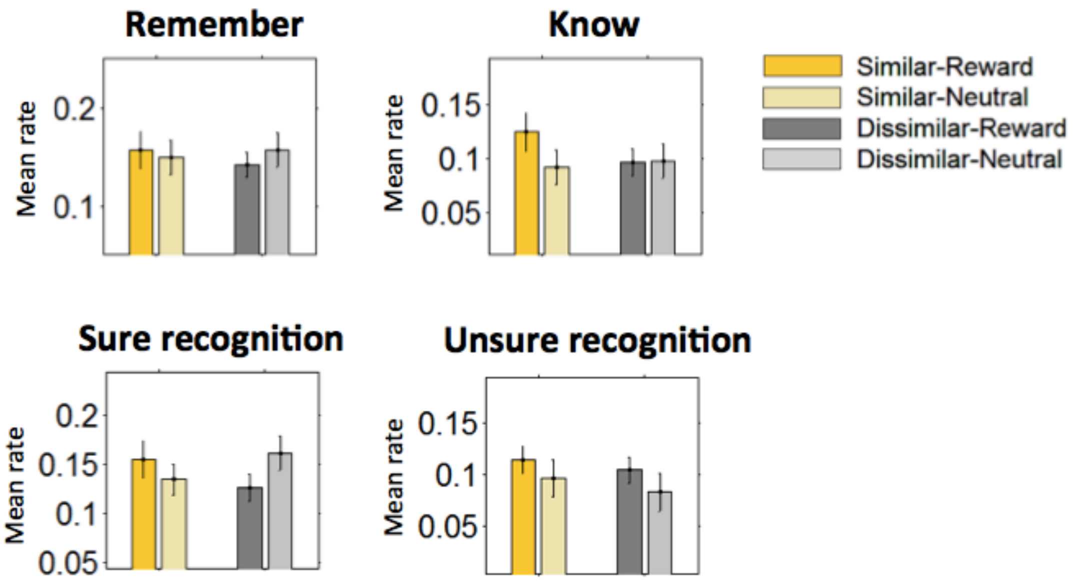

Supplementary Figure 2: Object-related neural responses. The use of a partial volume (optimized for coverage of the hippocampus and midbrain; A) limited our ability to examine object-related responses in areas like the visual cortex and object cortex. Widespread activation was observed contrasting the object presentations to baseline, including in regions like the parahippocampal cortex and perirhinal cortex (B). Even using an fMRI model that was optimized to find object-related effects that might support subsequent memory, we failed to find any evidence that the anterior DG/CA3 and SN/VTA results noted in the main results were fundamentally driven by neural responses to discrete objects (C; showing parameter estimates for hit>miss, split by similarity and valence conditions).

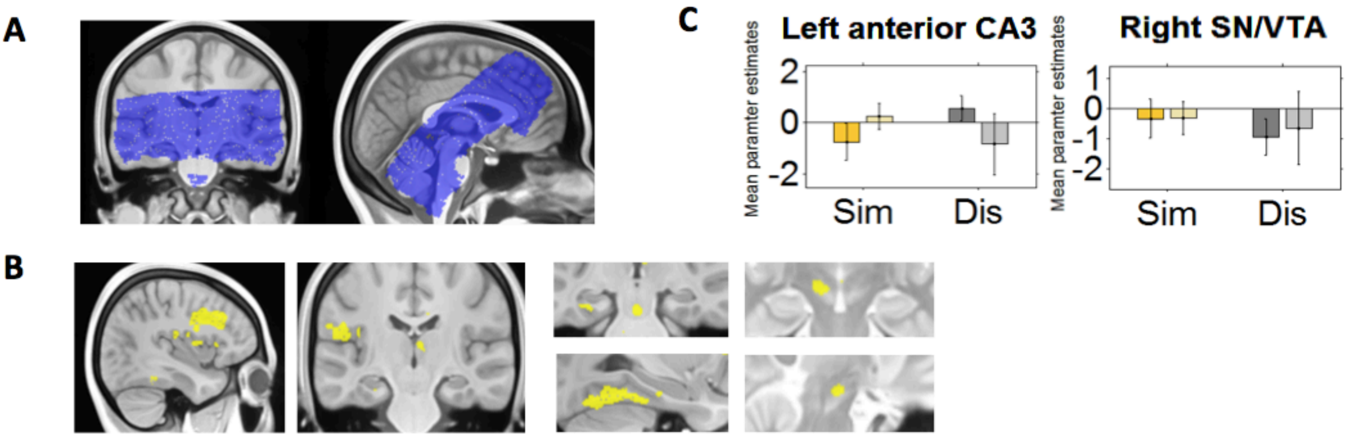

Supplementary Figure 3: Context-related SN/VTA activation is linked to context conditioning in the similar condition. Across all subjects, across all subjects, greater differences in the right SN/VTA response to the similar-reward context (as compared to the similar neutral) was correlated with greater reward-related RT speeding in the similar condition.

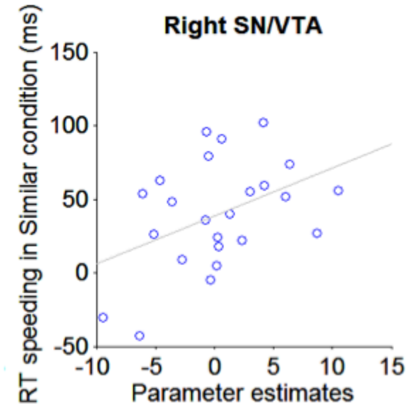

Supplementary Results

Further analyses to identify object-related activation that relate to subsequent memory

In the main fMRI model, context- and object-related regressors describing memory were allowed to compete for variance (with shared variance discarded). Such a model setup may have impaired our ability to identify object-related effects that related to memory. To further pursue the possibility that object-related activation may indeed be linked to subsequent memory, we built a separate fMRI model that *specifically* aimed to identify such object-related responses (i.e. rather than allowing context- and object-related regressors to compete for variance). This analysis effectively ignores context-related effects that may relate to memory, in order to optimise our ability to identify object-related effects. The model omitted the context-memory parametric modulators that had been included in the main fMRI model (see Materials and Methods for details on main model), and included 12s boxcar regressors for the presentation of the contexts epochs, collapsed over the four conditions (i.e. all condition in the 2x2, Similarity x Valence, design were collapsed into one regressor). We opted to collapse the context events into a single regressor (rather than modelling each context type separately), so as to reduce the extent to which the context and object regressors in each condition were correlated with each other (thus optimizing our ability to identify object-related neural findings that might explain the observed pattern of subsequent memory). The same eight object regressors were included in the model (as stick functions), describing objects by similarity, valence, and memory (recognition: hit vs miss) type. All other details of model setup were identical as with the main fMRI model reported in the main text (see Materials and Methods for full detail): error trials and the presentation of both informative feedback as well the non-informative feedback were included as regressors of no-interest, and additional covariates were included to capture residual artifacts related to movement, scanning session, heart rate and respiration. As in the main fMRI model, regressors relating to button presses were omitted from the model, since their inclusion would result in multicollinearity with the object regressors as a whole. As such, the object-related results reported reflect both processes that relate to object processing, as well as to the preparation and execution of motor responses.

First-level contrasts for the objects were fed into a 2x2x2 (similarity x valence x memory) second-level model. Using this new fMRI model, we again failed to find any object-related activation that related to subsequent memory (i.e. hit >miss), either as a function of context similarity, context valence, or an interaction between these two factors. We also directly tested if object-related responses were found in the regions that had previously shown a context-related memory effect (i.e. anterior DG/CA3 and SN/VTA; Figure 3A-B). We extracted parameter estimates from these regions, from the object-hit and object-miss contrasts in each of the four conditions (similarity x valence, 2x2). We then analysed the difference in the hit>miss parameter estimates with a 2x2 (similarity x valence) ANOVA, in order to examine if these regions additionally showed object-related responses that mirrored the pattern of memory effects. Using this

approach, we failed to find any main effects or similarity x valence interaction in either of these functional ROIs (all $p > 0.1$; Supplementary Figure 2C).

Again, our inability to identify object-related responses that relate to subsequent memory do not allow us to infer that object-related responses do not contribute to the observed pattern of memory effects. However, these results further indicate that the observed effects in the anterior DG/CA3 and SN/VTA (i.e. shown in Figure 3A-B) are unlikely to be fundamentally driven by discrete responses to individual objects themselves. Instead, the improved memory in the similar-reward condition is likely to be linked to the strength of the neural response in the DG/CA3 and SN/VTA in response to the entire context event as a whole.

<u>SN/VTA activation in response to the contexts is linked to reward-conditioning</u>

The asymmetry between the activation of the SN/VTA in the similar and dissimilar conditions motivated us to conduct further exploratory analyses. Given that the previous analysis has indicated stronger SN/VTA activity in response to the similar versus the dissimilar contexts, we investigated if the strength of the SN/VTA response was preferentially related to conditioning and memory performance in the similar condition. We extracted parameter estimates from SN/VTA ROIs (anatomically defined), from the four context-epoch contrasts themselves (i.e. *not* from the context-memory parametric modulators), and subjected these parameter estimates to correlational analyses. Reward-related differences in the right SN/VTA (anatomically defined) were found to correlate with the amount of reward-related RT speeding in the similar condition alone ($r = 0.426$, $p = 0.038$, Supplementary Figure 3; trend level correlation only for the left SN/VTA, $r = 0.276$, $p = 0.096$). No such correlations were observed in the dissimilar condition for either the left or right SN/VTA (both $p > 0.2$), or for the left or mid SN/VTA ROIs. Direct comparison of the correlation coefficients confirmed that the correlation between reward-related differences in RTs and reward-related differences in the right SN/VTA response was stronger in the similar condition as compared to the dissimilar (right: $z = 2.23$, $p = 0.026$ one-tailed; left: $z = 1.62$, $p = 0.053$ one-tailed). These results suggest that the selective SN/VTA responding in the similar-reward condition is related both to successful context conditioning in the similar condition, in addition to the memory effects that are noted in the main text.
